# Gap junction communication dynamics and bystander effects from ultrasoft X-rays

**DOI:** 10.1038/sj.bjc.6601686

**Published:** 2004-03-02

**Authors:** G O Edwards, S W Botchway, G Hirst, C W Wharton, J K Chipman, R A Meldrum

**Affiliations:** 1 School of Biosciences, The University of Birmingham, Edgbaston, Birmingham B15 2TT, UK; 2 Rutherford Appleton Laboratory, Chilton, Oxfordshire OX11 0QX, UK

**Keywords:** bystander effect, fluorescence redistribution, gap junction, ultrasoft X-rays

## Abstract

Gap junctions provide a route for small molecules to pass directly between cells. Toxic species may spread through junctions into ‘bystander’ cells, which may be exploited in chemotherapy and radiotherapy. However, this may be prevented by junction closure, and therefore an understanding of the dose-dependency of inhibition of communication and bystander effects is important. Low-energy ionising radiation (ultrasoft X-rays) provides a tool for the study of bystander effects because the area of exposure may be carefully controlled, and thus target cells may be clearly defined. Loss of gap junction-mediated intercellular communication between irradiated cells was dose-dependent, indicating that closure of junctions is proportional to dose. Closure was associated with hyperphosphorylation of connexin43. Inhibition of communication occurred in bystander cells but was not proportional to dose. Inhibition of communication at higher radiation doses may restrict the spread of inhibitory factors, thus protecting bystander cells. The reduction in communication that takes place in bystander cells was dependent on cells being in physical contact, and not on the release of signalling factors into the medium.

An increasing number of experimental observations reveal that toxic effects can be spread between cells in multicellular organisms even when neighbouring cells have not come into contact with the toxicant. This phenomenon is facilitated by several different cellular pathways, which are collectively known as the ‘bystander’ effect (Mothersill and Seymour, 2001).

It is easily appreciated how important it is to understand this phenomenon since the effects of toxicants might be underestimated if a direct dose relationship is assumed. This is of particular concern when considering the dose–response relationship and risk assessment of radiation. Radiotherapy is a common cancer treatment, and an accurate knowledge of dose–response relationships is vital to ensure successful treatment without endangering the patient. Furthermore, bystander effects in radiation therapy may augment the killing effects of radiation in tumour therapy (Chipman *et al*, 2003). Following radiation exposure, toxic factors might be spread or transferred by diffusion through aqueous media in organisms or cell cultures or may involve connexin-mediated gap junction intercellular communication (GJIC) (Mothersill and Seymour, 2001). Pinpointing the actual mechanism responsible for a bystander effect forms a major challenge.

The basic subunit of the gap junction is the connexin protein, and to date 21 different connexins have been cloned (Willecke *et al*, 2002). Six connexins form a hexamer around a central pore called a connexon, which is trafficked to the extracellular membrane (Evans and Martin, 2002). Gap junctions are formed when connexons on adjacent cells dock to form a pore between cells, allowing cytoplasmic continuity. The structure and function of gap junctions have recently been reviewed (Evans and Martin, 2002; Chipman *et al*, 2003). Gap junctions allow direct passage of molecules between cells, by a process known as GJIC. Closure of gap junctions has previously been described as involving changes in connexin phosphorylation (Lampe and Lau, 2000; Rivedal and Opsahl, 2001).

The bystander effect is of importance in cancer gene therapy (Molten, 1986) as GJIC mediates the transfer of gene products from transfected into nontransfected cells resulting in cell death. Transfection of cells with the herpes simplex virus thymidine kinase gene followed by treatment with the nucleotide analogue ganciclovir (GCV) results in phosphorylation of GCV. Phosphorylated GCV is incorporated into cells’ DNA terminating DNA replication, leading to cell death. This is enhanced in cells transfected with functional connexin genes as it is thought that phosphorylated GCV may pass through gap junctions (Tanaka *et al*, 2001). A current research approach to cancer treatment involves the combination of chemotherapy and radiotherapy (Patterson *et al*, 2003): if chemotherapeutic metabolites are required to pass from cell to cell to result in bystander killing, then knowledge of the effects of radiation on gap junctions is desirable. Gap junction intercellular communication has also been implicated in the inter-cell transfer of radiation effects (Azzam *et al*, 1998; Zhou *et al*, 2002). Bystander cells express the stress-inducible protein p21^Waf1^, micronucleus formation and p53 phosphorylation dependent on functional GJIC (Azzam *et al*, 2001). What makes it difficult to distinguish clearly between GJIC mechanisms and other mechanisms of transfer in cells exposed to chemicals or radiation is the lack of precise control over the regions of cells directly exposed. Radiation is ideally confined within a single cell's boundaries if gap junction transport is to be studied.

Low-energy ultrasoft X-rays provide an ideal source of radiation for this purpose because their properties allow spatial definition of exposed regions in a sample. Development of a laser plasma-generated ultrasoft X-ray source began in the late 1980s (Turcu *et al*, 1990, 1994). The source uses a system of excimer lasers, which produces a train of picosecond pulses. These are focused down to a 10 *μ*m spot onto a moving tape of a target material. The ablation of the material, which is heated typically to 10^7^ K, results in the thermal generation of X-rays. The target material can easily be changed and thus the system is capable of generating X-rays of different wavelengths. The material used for exposure of cells was copper, which gives rise to 1.1 keV 1.2 nm X-rays. Cells were grown on Hostaphan of 1 *μ*m thickness, and the penetration of the X-rays into the cells was less than 5 *μ*m (Goodhead and Thacker, 1977). The energy of the X-rays is so low that the interaction of the radiation with cellular components only takes place in cells on which the radiation directly impinges. The X-ray spectrum from the laser plasma source is sufficiently soft for long-range scattering to be very weak. This ensures that radiation of the surrounding medium is avoided so that radiolysis products from this source will not complicate the experiments.

Studies on the radiation bystander effect, which imply that GJIC plays a major role in spreading the effects of radiation, have used *α*-particle radiation (Azzam *et al*, 1998, 2001), which is high linear energy transfer (LET) radiation and will penetrate the full depth of a cell. The current study uses low LET radiation, and the tracks have an attenuation length of only 3.4 *μ*m (1/e), which ensures that the radiation will be fully absorbed within the cell.

To investigate the effects that radiation has on the function of gap junctions in directly irradiated and bystander cells, we have combined pulsed-laser plasma-generated ultrasoft X-rays (Turcu *et al*, 1990, 1994), micropositioning technology and photobleaching of single dye-loaded cells. We define a dose–response relationship and the spatial extent of the responses to localised irradiation in cultured cell populations. Changes in the phosphorylation of connexin43 (Cx43) have also been investigated.

## MATERIALS AND METHODS

### Cell culture

Rat liver epithelial cells (WB-F344, a kind gift from Professor WB Coleman) were cultured in improved minimum essential medium (Biosource International, Nivelles, Belgium) supplemented with 2 mM glutamine (Sigma Chemical Co., Poole, Dorset, UK), 5% v v^−1^ foetal calf serum (Labtech International, Ringmer, East Sussex, UK) and 50 *μ*g ml^−1^ gentamicin (Sigma). Cultures were incubated at 37°C in 5% CO_2_/95% air. Cells were passaged every 3–4 days, when confluent, via trypsinisation. These cells express Cx43 and are competent in GJIC (Stock *et al*, 1998).

### Irradiation of cells with ultrasoft X-rays

In order to expose cells to ultrasoft X-rays, they were cultured in chambers constructed from 35 mm diameter glass rings fitted with a 1 *μ*m thick Hostaphan base (Hoechst). The X-rays were generated from a laser plasma source as described previously (Meldrum *et al*, 2002). A 25 *μ*m × 3 mm slit was manufactured in a disc of stainless steel of 13 *μ*m thickness. This disc was fitted into the wall of the chamber directly above the point of the laser plasma. The stainless steel is completely opaque to the 1.1 keV X-rays. An aluminium filter (3 *μ*m thick) placed between the X-ray source and the X-ray outlet excludes all visible and UV light.

Dishes of cells were placed in a chamber holder that could be seated in six predetermined positions on the X-ray source and exposed to 1, 3 or 5 Gy soft X-rays. It has previously been shown that a dose of 5 Gy caused extensive cytotoxicity to V79-4 Chinese hamster ovary cells, through a delayed mechanism resulting from DNA damage and measurable by colony formation at 7 days postirradiation (Botchway *et al*, 1997). Both the X-ray source stage and the confocal microscope stage were modified by the addition of a series of recesses, allowing the chamber holder to be positioned by means of triangulation between three points. The stage on the confocal microscope was controlled by a computer running an ‘in-house’ program (programmed on LabVIEW™). Six regions of illumination were defined, detected on Gaffchromic film, and the confocal stage was calibrated using this so that the computer could send the stage to an exact region of illumination. By doing this, it was possible to reposition the stage to investigate both directly irradiated cells and cells a known distance from the illuminated region.

### GapFRAP

Gap junction intercellular communication was monitored using the GapFRAP (fluorescence redistribution after photobleaching via gap junctions) technique (Frame and de Feijter, 1997; Cotrina *et al*, 1998). Confluent cultures of cells were washed twice with phosphate-buffered saline (PBS) and placed in medium containing 10 *μ*M 5,6-carboxyfluorescein diacetate (CFDA) (Molecular Probes, Cambridge Biosciences, Cambridge, UK). These were incubated for 15 min at 37°C, after which they were washed three times in PBS, and placed under supplemented culture medium prior to irradiation. Cells were examined with a × 20 objective, and fluorophores excited with a 25 mW argon-ion laser (488 nm). A field of view containing confluent fluorescing cells was selected and an image captured (‘prebleach’). Using the zoom function on the confocal microscope, a single cell was selected and five passes of the argon-ion laser, operating at full power (<1 mW at the microscope stage), bleached the dye in this cell. The microscope was returned to the previous configuration and an image, ‘postbleach’, was captured. At 4 min postbleaching, a final image was captured. Treatments were replicated 9–11 times. The degree of redistribution of fluorescence into the photobleached cell was determined using the ScionImage computer program (Scion Corporation, Frederic, Maryland, USA).

### Phosphorylation of Cx43

Whole dishes of cells were exposed to 5 Gy, 1.1 keV ultrasoft X-rays. Cells were scraped into PBS, pelleted, frozen in liquid nitrogen and stored at −70°C. As a positive control for hyperphosphorylation (Rivedal and Opsahl, 2001; Yang *et al*, 2001), dishes of cells were treated with 20 nM 12-*O*-tetradecanoylphorbol-13-acetate (TPA) for 15 min and collected in the same manner as irradiated cells. For analysis, pellets were thawed and resuspended in 100 *μ*l lysis buffer (1% w v^−1^ SDS, 1 mM sodium vanadate, 10 mM Tris-HCl, pH 7.4) and disrupted by passing repeatedly through a 25G syringe. Total protein in a 10 *μ*l aliquot was determined by the BioRad *D*_*c*_ assay (BioRad, Hemel Hempstead, Hertfordshire, UK), and compared against a standard curve of bovine serum albumin (Sigma). An aliquot of sample containing 15 *μ*g total protein was mixed at a ratio of 1 : 1 with reducing sample buffer (0.125 M Tris-HCl, pH 6.8; 20% w v^−1^ glycerol; 5% w v^−1^ SDS; 5% v v^−1^ *β*-mercaptoethanol; 0.1% w v^−1^ bromophenol blue) and incubated for 10 min at room temperature. Electrophoresis was carried out on 12.5% SDS–polyacrylamide gels (Laemlli, 1970). Control gels were stained with Coomassie blue to assess protein separation. Proteins were transferred to Hybond-C Extra membrane (Amersham Biosciences, Little Chalfont, Buckinghamshire, UK), which was blocked for 1 h at room temperature with 5% w v^−1^ skimmed milk powder in TBST (10 mM Tris-HCl, pH 8.0; 150 mM NaCl; 0.05% Tween-20), and probed for 1 h with a mouse anti-rat-Cx43 primary antibody (BD Pharmingen, Cowley, Oxfordshire, UK). Blots were then stained with a horseradish peroxidase-conjugated goat anti-mouse secondary antibody (DakoCytomation, Ely, Cambridgeshire, UK) and detected by the enhanced chemiluminescence method (Amersham).

### Assessment of cell membrane integrity

Dishes of cells were exposed to 1, 3, 5 and 10 Gy soft X-rays and incubated under standard growth conditions for 30 min and 2 h. Following incubation, the culture medium was collected and assayed for lactate dehydrogenase (LDH) activity (Moldeus *et al*, 1978). To a cuvette, 530 *μ*l potassium phosphate buffer was added (66 mM K_2_HPO_4_, 34 mM KH_2_PO_4_, pH 7.0), along with 50 *μ*l 23 mM sodium pyruvate. The supernatant from the cell cultures (400 *μ*l) was added. Finally, 10 *μ*l of a 12 mM NADH solution (freshly prepared and stored on ice) was added, and cuvettes were mixed by inversion. The decrease in absorption at 340 nm over 30 s was measured, giving an indication of LDH activity. Cells were scraped from Hostaphan membranes, lysed in lysis buffer, and solids were pelleted out by microcentrifuging samples for 1 min at 13 000 r.p.m. Total protein was measured with the BioRad *D*_*c*_ assay. Controls consisted of untreated cells and cells treated with 0.5% v v^−1^ Triton X-100. The latter provided a positive control for 100% LDH leakage. Data were expressed as relative LDH activity, calculated by dividing LDH activity by the total protein per dish. Treatments were carried out in triplicate and differences analysed using the *t*-test.

## RESULTS

### Spatial irradiation of cells

Figure 1A

**Figure 1:**
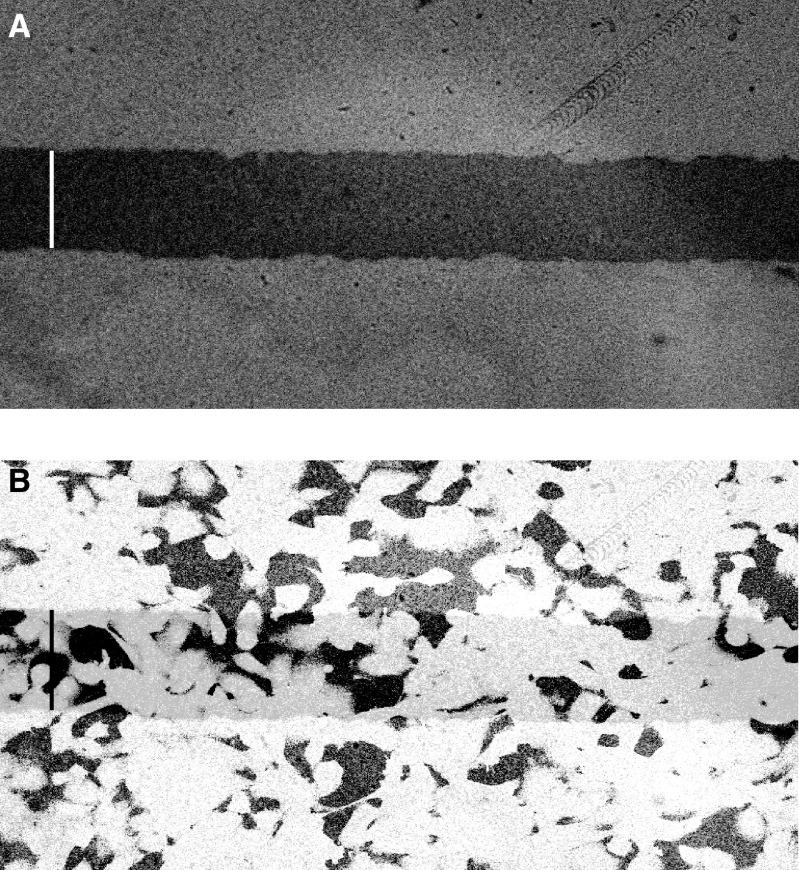
(**A**) Micrograph of Gaffchromic film illuminated with 400 Gy ultrasoft X-rays, demonstrating a sharply defined region of irradiation. Bar represents 100 *μ*m. (**B**) Fluorescently labelled cells were overlaid on the film to demonstrate how many cells are exposed to ultrasoft X-rays and confirm that translation from the X-ray source to the confocal microscope relocated irradiated cells accurately. Bar represents 100 *μ*m.

shows the image obtained when Gaffchromic film was exposed to 400 Gy of 1.1 keV X-rays, through a 100 *μ*m slit. The area of cells irradiated in a confluent culture can be determined by overlaying the cell culture dish on the exposed film (Figure 1B). If used in conjunction with the micropositioning and relocation system, it is possible to confirm exactly where and how many cells were irradiated. This helps to choose a cell to photobleach for GapFRAP analysis as it enables cells to be located both within the exposed region and at defined distances from exposed cells. As well as providing precise spatial control over cells irradiated, the pulsed nature of the ultrasoft X-ray source allows the dose of X-rays to be controlled, and as the system is capable of delivering 3 Gy s^−1^, a substantial dose may be given in a short time.

### GapFRAP analysis

**Figure 2:**
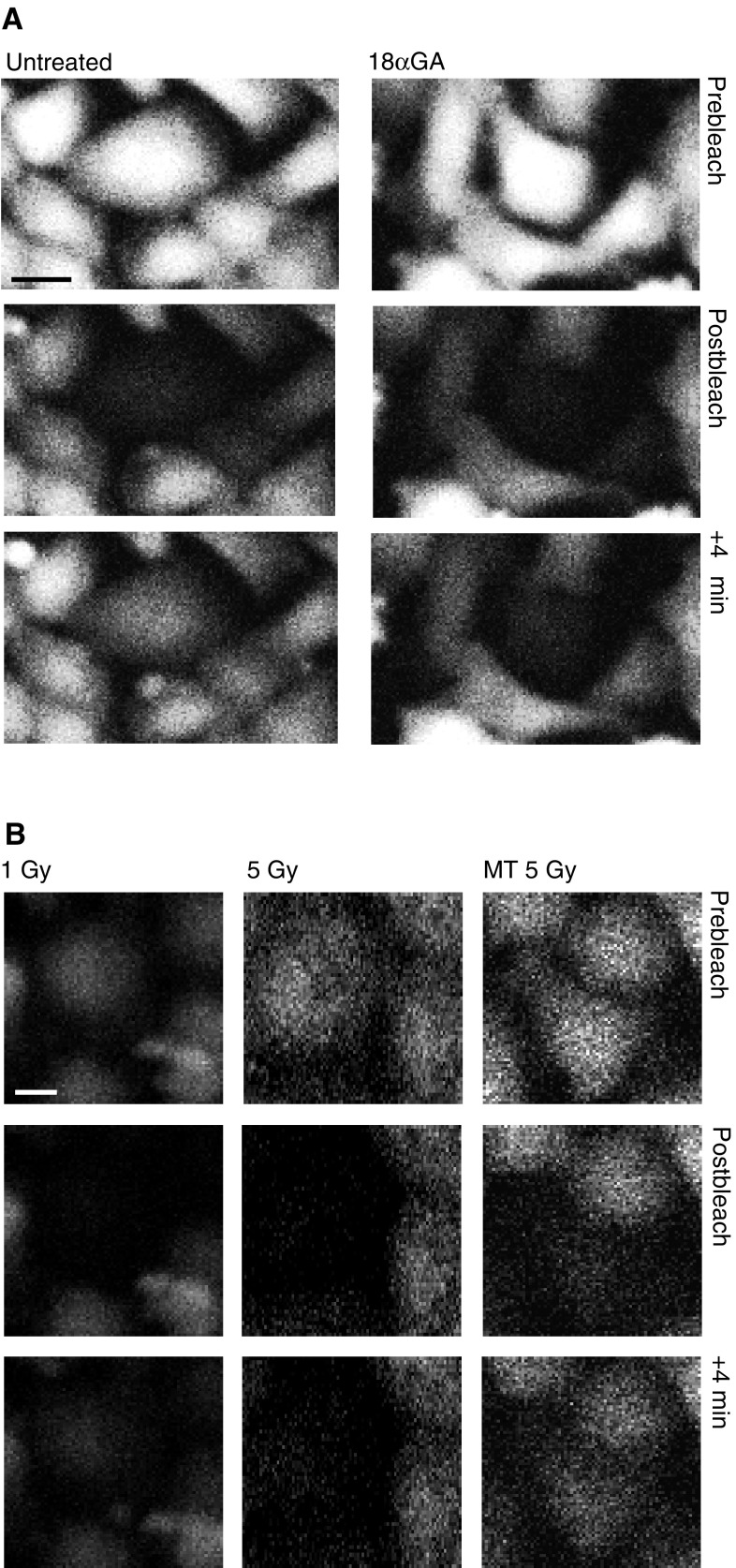
Confocal fluorescence images of WB-F344 cells demonstrate the GapFRAP assay. Prebleach, postbleach and +4 min postbleach are shown in (**A**) untreated cells and cells treated with 25 *μ*M 18*α-*glycyrrhetinic acid (18*α*GA; gap junction inhibitor) and (**B**) cells directly exposed to 1 Gy ultrasoft X-rays (1 Gy), cells directly exposed to 5 Gy ultrasoft X-rays (5 Gy) and unirradiated cells treated for 15 min with media transferred after 15 min from cells that had been exposed to 5 Gy ultrasoft X-rays (MT 5 Gy). Bar represents 10 *μ*m.

Glycyrrhetinic acid was used as a positive control as it is known to inhibit GJIC (Goldberg *et al*, 1996; Tare *et al*, 2002). This provides a negative index against which the extent of fluorescence redistribution after photobleaching in irradiated cells can be compared. Fluorescence redistribution in untreated cells provides a positive index of GJIC. Figure 2A

shows the fluorescence redistribution 1 and 4 min after photobleaching of a single cell in untreated cells and cells treated with 25 *μ*M 18*α*-glycyrrhetinic acid for 30 min. To derive an index of fluorescence redistribution, fluorescence intensity was measured in the unbleached cell and the fluorescence intensity 4 min after photobleaching is expressed as a percentage of the initial fluorescence content of the cell. In the untreated cells, the fluorescence recovered to approximately 20% of the initial value within 4 min, but in the 18*α*-glycyrrhetinic acid-treated cells this only recovered to 6% of the initial value. A baseline comparison was derived from sets of control untreated cells for each individual experiment. The value of fluorescence redistribution in irradiated cells is henceforth described in terms of a percentage of the redistribution that takes place in the matched set of untreated cells.

### Effects of ultrasoft X-irradiation on intercellular communication

Cells were loaded with CFDA and irradiated through the 100 *μ*m slit with 1, 3 and 5 Gy ultrasoft X-rays. GapFRAP was carried out at 3 and 15 min postirradiation (Figures 2B

**Figure 3:**
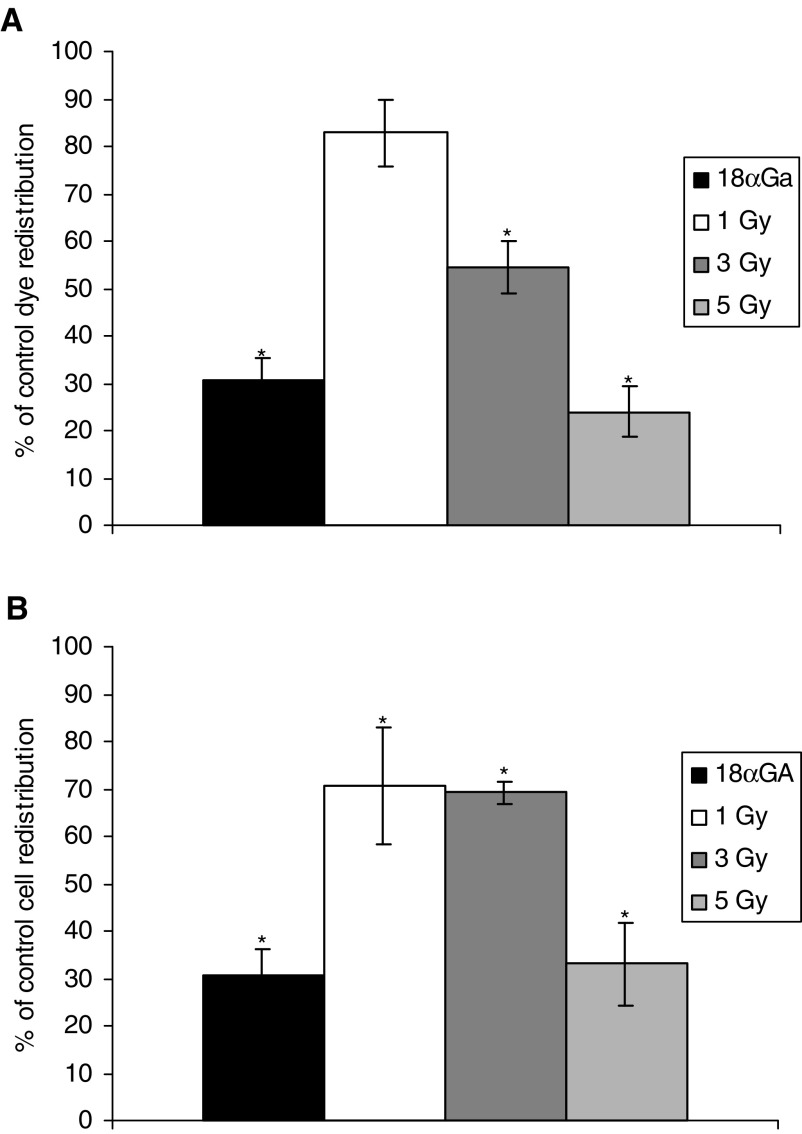
Dose–response relationship between dose of ultrasoft X-rays and gap junction communication (rate of redistribution of fluorescence in the GapFRAP assay) in directly irradiated WB-F344 rat liver epithelial cells at (**A**) 3 min and (**B**) 15 min postexposure, compared to cells treated with 25 *μ*M 18*α-*glycyrrhetinic acid (18*α*GA; gap junction inhibitor). Results are expressed as % communication compared to control (untreated) cells (5.022 U min^−1^±0.275). Bars show standard error of the mean.

, 3A,B). The fluorescence in cells 4 min postbleaching was recorded. At 3 min postirradiation, exposure of cells to 1 Gy reduced the recovery of fluorescence in the photobleached cells to 82.8±7.1% (*P*=0.057) of initial values, 3 Gy to 54.5±5.5% (*P*<0.01) and 5 Gy to 24.0±5.4% (*P*<0.001). The inhibition of intercellular communication by 5 Gy ultrasoft X-rays was comparable in magnitude to that induced by 25 *μ*M 18*α*-glycyrrhetinic acid. At 15 min postirradiation, the inhibitory effects were still apparent and became statistically significant at 1 Gy but were similar or reduced (15 min compared to 3 min) at 3 and 5 Gy.

### GapFRAP analysis of bystander cell communication

**Figure 4:**
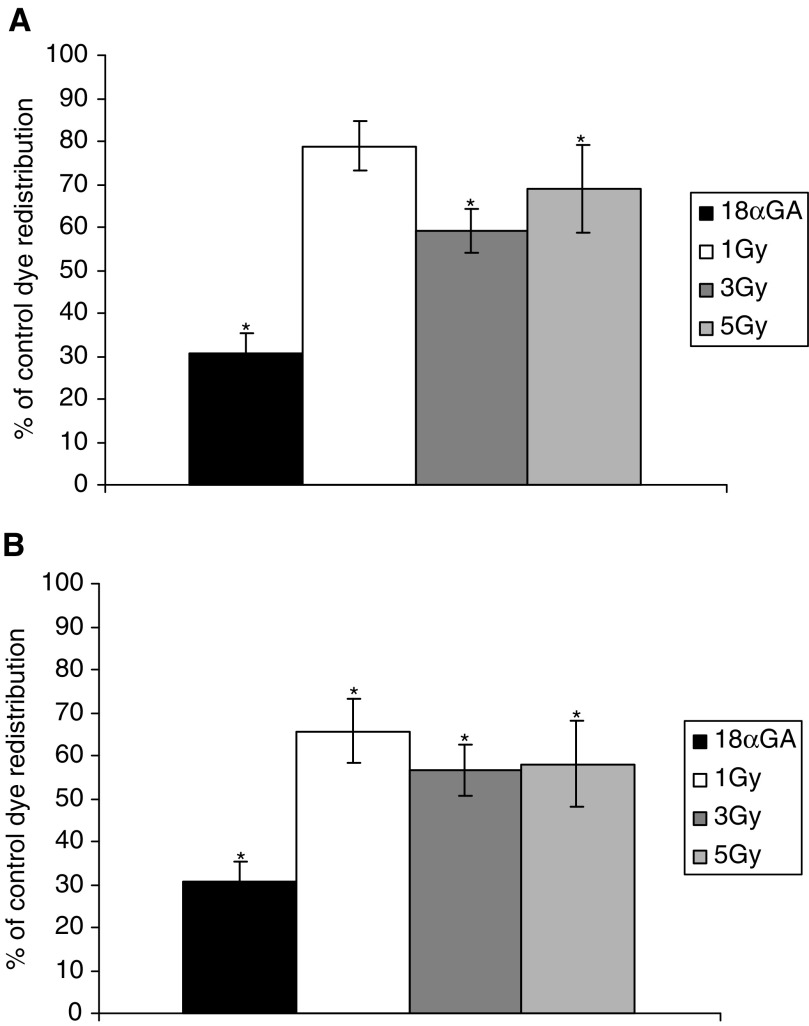
Dose–response relationship of ‘bystander’ WB-F344 rat liver epithelial cells confluent with irradiated cells at (**A**) 3 min and (**B**) 15 min postexposure, compared to cells treated with 25 *μ*M 18*α-*glycyrrhetinic acid (18*α*GA; gap junction inhibitor). Results are expressed as % communication compared to control (untreated) cells (5.022 U min^−1^±0.275). Bars show standard error of the mean.

Cultures of confluent cells were irradiated through the 100 *μ*m slit, and GapFRAP analysis of unirradiated ‘bystander’ cells was carried out at a distance of 100 *μ*m from the irradiated cells. Reduction of communication between these cells was detected but was of a lesser magnitude at 3 min postirradiation than in the directly irradiated cells (Figure 4A

). Communication in bystander cells after treatment with 1 Gy ultrasoft X-rays was reduced to 78.9±5.9% (*P*<0.05) of that in untreated cells, 3 Gy to 59.3±5.1% (*P*<0.01) and 5 Gy to 69.0±10.0% (*P*<0.01). This illustrates a lack of a proportional relation with the dose of irradiation in bystander cells. A slight further suppression of intercellular communication was evident at 15 min postirradiation (Figure 4B), sufficient to make the effect of 1 Gy exposure statistically significant.

### Studies on the potential transfer of effects via the medium

**Figure 5:**
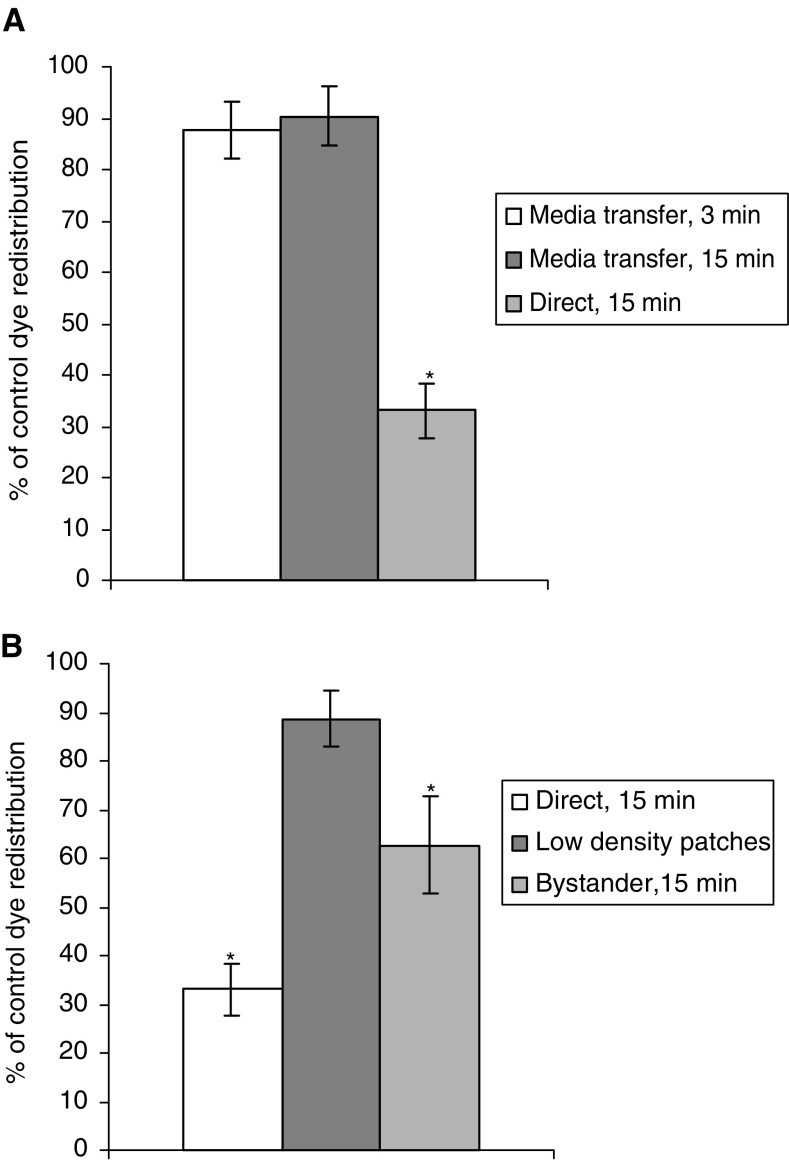
GapFrap in cells nonconfluent with irradiated cells: results are expressed as % communication compared to control (untreated) cells (5.022 U min^−1^±0.275). (**A**) Within the time domain of the experiment, no transfer of inhibitory molecules occurred by diffusion through the extracellular environment. (**B**) Confirmation that cells need to be confluent for radiation-induced inhibition of GJIC to occur. Low-density patches of cells were not confluent with directly irradiated (target) cells, and bystander cells were confluent with target cells.

To establish if the inhibition of GJIC in bystander cells could occur via an intercellular signal released into the medium, two further experiments were carried out. Whole dishes of confluent cells were exposed to 5 Gy ultrasoft X-rays and the medium transferred to dishes of nonirradiated confluent cells, 3 and 15 min postirradiation. Recipient cells were assayed for GJIC using GapFRAP up to 15 min after addition of medium from irradiated cells. GapFRAP demonstrated that communication in the recipients was not significantly different from that observed in untreated cells (Figure 5A

).

To investigate if a medium-borne factor could exert an effect on cells in close proximity to (but not in contact with) irradiated cells, cultures were seeded in 3 ml medium in growth chambers at 10^4^ cells ml^−1^ and cultured for 24 h. These conditions provided small clusters of cells isolated from each other. Samples were then irradiated with 5 Gy ultrasoft X-rays through the 100 *μ*m slit, and the micropositioning system employed so that GapFRAP could be carried out on clusters of nonirradiated cells approximately 100 *μ*m from (yet not confluent with) the irradiated cells. A transfer of effects on GJIC was not observed under these conditions (Figure 5B).

### Phosphorylation of Cx43

**Figure 6:**
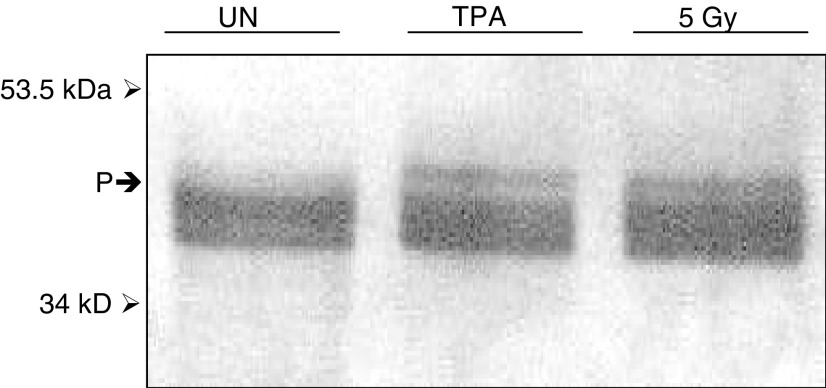
Western blot showing hyperphosphorylation of Cx43 in WB-F344 cells exposed to 5 Gy ultrasoft X-rays (5 Gy) and 20 nM TPA. (P) Demarcation of the band showing phosphorylation in irradiated and TPA-treated cells and the absence of such a band in untreated cells (UN).

Gap junction closure involves hyperphosphorylation of connexins and the phosphorylation of Cx43 can be detected by altered electrophoretic mobility of the proteins on a Western blot. The phorbol ester and tumour promoter TPA, which is known to induce Cx43 hyperphosphorylation (Rivedal and Opsahl, 2001; Yang *et al*, 2001), was used as a positive control to compare with cells irradiated with the ultrasoft X-rays. Western blotting of SDS–polyacrylamide gels revealed a difference in phosphorylation of Cx43 in WB-F344 cells treated with 20 nM TPA for 15 min and cells irradiated with 5 Gy ultrasoft X-rays, 15 min postexposure compared to controls. In Figure 6

, a band of reduced electrophoretic mobility is seen in isolates from cells treated with TPA and 5 Gy ultrasoft X-rays. This band indicates hyperphosphorylation of Cx43 in these cells.

### Effects of soft X-rays on membrane permeability

In order to confirm that the observed effects on GJIC were due to cells responding to radiation and not due to secondary effects of loss of membrane integrity and cell death at the time points investigated, membrane permeability was assessed by the leakage of LDH and was found not to be significantly reduced compared to untreated cells (*P*=0.27) over the times of experiments carried out, nor at 2 h postexposure. As a positive control, membranes were permeabilised with Triton X-100 and LDH leakage was significantly greater than in untreated cells (*P*=0.0004).

## DISCUSSION

We have developed and combined ‘state-of-the-art’ techniques in laser plasma X-ray generation, micropositioning and laser photobleaching in biological cells to investigate the effects of ionising radiation on intercellular gap junctions. The experiments illustrate how ionising radiation inhibits GJIC in a dose-dependent manner in directly irradiated cells. Inhibition of GJIC is effective at least up to 15 min after exposure to ultrasoft X-rays, without a loss of membrane integrity.

Phosphorylation of connexins controls the assembly, operation and degradation of the gap junctions (Lampe and Lau, 2000). When WB-F344 cells were exposed to the tumour promoter TPA, a loss of GJIC was observed and this was associated with increased hyperphosphorylation of Cx43 (Rivedal and Opsahl, 2001; Yang *et al*, 2001). This was attributed to the TPA-mediated activation of protein kinase C (Ren *et al*, 1998), but interaction with the mitogen-activated protein kinase (MAP kinase) pathway may also be necessary (Rivedal and Opsahl, 2001). These changes in phosphorylation appear to result in internalisation of connexins into the cytoplasm, disassembling the gap junctions and cutting off communication between cells (Yang *et al*, 2001). These changes are also observed when cells enter mitosis (Xie *et al*, 1997) as well as when they enter into apoptosis (Huang *et al*, 1998, 2001; Koffler *et al*, 2000). V-src tyrosine protein kinase and casein II kinase have also been shown to phosphorylate certain connexins (Lampe and Lau, 2000; Yin *et al*, 2001).

Our studies show that the inhibition of communication by ultrasoft X-rays appears to involve the hyperphosphorylation of the gap junction protein Cx43 in a manner similar to that induced by the tumour-promoting agent TPA. The positive and negative regulation of gap junction communication has previously been demonstrated to be associated with phosphorylation of connexin on serine/threonine and tyrosine residues (Cooper *et al*, 2000). The regulation of Cx43 trafficking to the membrane, subsequent formation of gap junction plaques, single channel behaviour and degradation by phosphorylation are represented in many cell types. These events have also been shown to be TPA sensitive and regulated by PKC (Lampe and Lau, 2000). It appears that ionising radiation may have effects similar to TPA and that the phosphorylation of Cx43 is affected in a similar manner.

Our results demonstrate that a signal causing a reduction in GJIC passes from directly exposed cells to confluent ‘bystander’ cells within a distance of 100 *μ*m from the irradiated cells. This distance represents about five cell diameters. When GJIC is inhibited by radiation in target cells, a reduction in communication (rather than a complete inhibition) still alters the transfer of a damaging signal to bystander cells, albeit in reduced quantities explaining the nonlinear dose response.

Studies using cultured cells have described observations that implicate a factor that is produced by irradiated cells and transferred through the culture medium to nonirradiated cells (Little, 2000; Zhou *et al*, 2002). The medium-borne factor requires that high numbers of cells be exposed to irradiation before an effect is seen upon recipient cells or that the factors released into the medium are sufficiently concentrated, that is, medium transfer effect is dependent on the ratio of cell number to medium volume.

The medium transfer experiments we carried out suggest that the signal is not transferred via the medium (at least not within the time frame of these studies). More importantly, the results of experiments that used nonconfluent patches of cells showed that no reduction of GJIC was seen in patches of cells at a distance of 100 *μ*m and separate from irradiated cells. This confirmed that a contiguous link of bystander cells with irradiated cells has to exist before the immediate postirradiation inhibition of GJIC in the former is observed.

In the studies using *α*-particles, the participation of gap junction transfer was established by using inhibitors of gap junction communication. End points that have been measured in the *α*-particle studies are micronucleus formation, expression of the stress-inducible protein p21 ^Wafl^ and phosphorylation of p53 (Azzam *et al*, 2001). These have all been seen to occur in more cells than would be expected from direct exposure alone (Belakov *et al*, 2001), and were reduced when cells were incubated with GJIC inhibitors (Azzam *et al*, 2001).

By targeting the nuclei of cells with a *α*-particle microbeam, mutations at the CD59 locus were seen to accumulate. When the *α*-particle microbeam was focused on the cytoplasm of a cell, free radicals were generated and gave rise to mutations in the nucleus of the same cell (Wu *et al*, 1999), but these free radicals do not appear to be transferred by the gap junctions to bystander cells (Zhou *et al*, 2000). This is a reasonable conclusion as the free radical OH^•^ diffuses no further than 4 nm before reacting with biomolecules (Chapman *et al*, 1973). Furthermore, gap junctions can demonstrate charge selectivity, although most gap junction channels favour the transfer of positively charged dyes and ions by a factor of 2–5 (Veenstra, 1996).

Previous reports have demonstrated that radiation-induced stress may propagate between directly irradiated and unexposed cells through gap junctions (Azzam *et al*, 2001; Huang *et al*, 2003). The findings reported on in this paper add to these publications, demonstrating that radiation may also inhibit GJIC, perhaps constituting a protective mechanism to prevent the propagation of radiation-induced stress. As radiation may disrupt GJIC, radiation type and dose regimes need to be carefully chosen when combining chemotherapy and radiotherapy in cancer treatment in order to allow the spread of cytotoxic metabolites (Patterson *et al*, 2003).

In conclusion, the techniques employed herein have enabled us to establish that Cx43 phosphorylation is associated with loss of GJIC induced by ultrasoft X-ray exposure. Bystander cells also exhibit reduced GJIC with an apparent nonlinear dose dependency, reflecting the role of GJIC itself in the propagation of the inhibitory signal(s) of unknown identity. It should be noted that the level and nature of the bystander effects are likely to be dependent on the cell type (Hall, 2003), possibly due to different connexin expression profiles.
